# Posttraumatic growth following culturally grounded intervention among tribal young women with dissociative disorders in Sikkim: an interpretative phenomenological analysis

**DOI:** 10.3389/fpsyg.2026.1927590

**Published:** 2026-09-01

**Authors:** Devendra Singh

**Affiliations:** DCV Institute of Indology, Lucknow, India

**Keywords:** breathwork, culturally grounded intervention, dissociative disorders, India, interpretative phenomenological analysis, posttraumatic growth, tribal communities, TRIBE-HEAL

## Abstract

**Background:**

Trauma recovery among tribal adolescent girls and young women in Northeast India remains poorly understood, particularly for those with dissociative disorders. Existing frameworks rely on Western paradigms that do not account for the cultural, spiritual, and environmental realities of tribal communities.

**Methods:**

Using Interpretative Phenomenological Analysis (IPA), this study examined the lived healing experiences of three young women from Sikkim who had completed a culturally grounded intervention comprising Pranayama, Ana Pana Sati meditation, and raga-based music therapy in 2022. Long-form semi-structured follow-up interviews were conducted in 2025.

**Results:**

Analysis yielded four superordinate experiential domains: (1) Psychological and Emotional Transformation, (2) Sociocultural and Systemic Trauma Context, (3) Embodied and Relational Pathways to Healing, and (4) Non-Linear Recovery and Ongoing Vulnerability. Participants described significant posttraumatic growth including emotional liberation, identity reconstruction, and altruistic orientation, alongside persistent sociocultural stressors and episodic relapse.

**Conclusion:**

These findings informed the TRIBE-HEAL model (Trauma Resilience Integration using Breathwork and Expressive Healing Approaches in Local contexts), a culturally grounded trauma-recovery framework addressing the biopsychosocial-spiritual dimensions of healing in marginalized tribal settings. TRIBE-HEAL is proposed as an exploratory, hypothesis-generating framework derived from a small idiographic sample; it has not been independently validated and requires further testing before clinical application.

## Introduction

Trauma among marginalized tribal young women in Northeast India is shaped by compounding adversities: gender-based violence, stigma, poverty, institutional neglect, and social marginalization, all of which increase vulnerability to dissociative disorders (Chaturvedi et al., 2010; Jayan et al., 2025; Varshney et al., 2024). Despite this, how these individuals interpret their symptoms, seek help, and experience recovery within their cultural, relational, and environmental frameworks remains largely undocumented.

The existing literature on dissociation and trauma recovery is predominantly shaped by Western paradigms prioritizing cognitive or psychotherapeutic approaches (Herman, 1992; van der Kolk, 2014). Emerging scholarship highlights the importance of sociocultural factors, embodied self-regulation, and community-integrated resilience, particularly for marginalized groups (Gee et al., 2023; Jeste et al., 2025). In tribal settings with limited formal mental health access, young women rely on culturally embedded practices such as breathwork, music, spiritual rituals, and relational support (Varshney et al., 2025). Yet no study has systematically examined how these practices facilitate psychological transformation or posttraumatic growth in tribal young women with dissociative disorders.

In many rural and tribal regions in India, dissociative symptoms are interpreted through spiritual or possession frameworks, complicating early identification and trauma-informed care (Varshney et al., 2022). No trauma-informed, culturally grounded model currently exists to explain how healing unfolds in these communities. Specifically, while prior work from this research programme has characterized barriers to diagnosis and the physiological correlates of a breathwork-based intervention in this population (Varshney et al., 2022, 2024, 2025), none of these studies examined how affected young women themselves subjectively experience and organize sustained posttraumatic growth after treatment. It is this first-person, experiential gap that the present study addresses.

This study addresses this gap by examining: How do adolescent girls and young women from tribal communities in Sikkim who have experienced dissociative disorders describe their posttraumatic growth following culturally grounded healing interventions, and how do these experiences inform the development of a trauma-informed behavioral health model?

Using IPA, the study explores the lived experiences of three young women who participated in Pranayama, meditation, and raga-based therapeutic modalities. Their narratives illuminate the interplay of sociocultural oppression, embodied distress, relational safety, and identity evolution in their healing. These findings informed the development of TRIBE-HEAL (Trauma Resilience Integration using Breath-work and Expressive Healing Approaches in Local contexts), a culturally attuned trauma-recovery framework grounded in both lived experience and behavioral medicine principles.

## Materials and methods

### Study context

This study was conducted in Sikkim, India, a predominantly tribal region where adolescent girls and young women frequently encounter intersecting forms of trauma including gendered stigma, caste-linked discrimination, community neglect, and limited access to trauma-informed mental health care. Participants were initially recruited in 2022 from two government hospitals (Singtam District Hospital and New STNM Hospital, Gangtok) through purposive sampling by psychiatrists who had diagnosed them with dissociative (conversion) disorders. All three had completed the culturally grounded intervention (Pranayama, meditation, and raga-based music interventio) in 2022 and were re-contacted for long-term follow-up interviews in November 2025.

### Sample and participants

IPA emphasizes idiographic depth, privileging small, information-rich samples (Smith et al., 2009). This study included three adolescent and young adult female participants, consistent with established IPA guidelines recommending 3–6 participants for rigorous idiographic analysis. As shown in Table 1, three adolescent girls and young women with clinically diagnosed dissociative disorders from tribal and marginalized communities in Sikkim participated in this study. As is standard for IPA, the sample size follows Smith et al.’s (2009) guidance of three to six participants for a single idiographic study, and, consistent with this epistemology, findings are intended to offer theoretical transferability rather than statistical generalizability to the broader tribal population. Inclusion criteria were: clinically diagnosed dissociative (conversion) disorder (per ICD-10 criteria, applied by the referring psychiatrist); completion of the 2022 Pranayama (breath-work) and raga-based music intervention; ability to participate in-depth in Nepali, Hindi, or English; and willingness to provide informed consent for longitudinal follow-up.

**TABLE 1:** Demographic characteristics of the participants.

Case Id	Gender	Age	Education	SES	Occupation	Marital status	Domicile
P1	Female	21	Post-Graduate Diploma	Middle Class	Student/Online business	Single	Rural
P2	Female	27	Under-graduate Diploma	Lower Class	Student/actively looking for a job	Engaged	Rural
P3	Female	33	Post-Graduate	Middle Class	Working	Married	Rural

P1 (age 21) was initially hospitalized at 17 after a suicide attempt following dissociation, depression, aggression, and childhood sexual abuse trauma. After Pranayama and Ana Pana Sati meditation, she recovered significantly. At follow-up, she was completing a postgraduate diploma internship, managing an online business, and practicing breath-work consistently.

P2 (age 27) was a 23-year-old trainee with visual and locomotor disabilities, severe childhood physical and sexual abuse, and years of ineffective ritual-based treatments. Following breath-work, music and arts therapy intervention she improved markedly. By follow-up she had completed a Life Skills Diploma, enrolled in *Katha Vachak* (spiritual preacher) training, and centered her daily routine around meditation, mantra chanting, and flute-based raga practice.

P3 (age 33) was a 29-year-old lab technician with dissociative disorder marked by panic attacks, emotional numbing, memory gaps, and occupational impairment. She improved after intervention, married, and was employed in the state Health Department’s mobile clinic. She experienced a relapse related to family conflict but stabilized after relocating and resuming breathwork and meditation.

### Data collection

Ethical approval was obtained from the Institutional Ethics Committee of New STNM Hospital, Gangtok, and written informed consent was obtained from all participants. Long-form semi-structured interviews of 90–120 min were conducted in 2025, exploring participants’ memories of the intervention, healing processes, sociocultural contexts, embodied experiences, and perceptions of long-term change. Interviews were audio-recorded, transcribed verbatim, and translated into English where needed. Table 2 illustrates the variety of questions in the questionnaire.

**TABLE 2:** Sample of interview questions.

Q No	Interview question
1	It has been some time since your healing journey began. How do you see yourself and your life now compared to before the intervention?
2	During the difficult times, what helped you to keep going or find some inner strength?
3	What changes have you noticed in your body, breath, or emotions since practicing the techniques like Pranayama, music, or art therapy?
4	If another girl from your community went through something similar, what would you want her to know from your experience?

### Data analysis

Data were analyzed using IPA following Smith et al. (2009). Analysis began with thorough transcript immersion, followed by line-by-line initial coding to capture experiential statements, emotional nuances, and meaning-laden expressions. Emergent themes were then articulated in participants’ own language, retaining an idiographic focus before advancing to interpretative generalizations. Themes were organized into superordinate domains informed by conceptual interrelations and theoretical acuity, while consistently revisiting original transcripts to maintain fidelity. Reflexive memos were maintained throughout to track interpretive choices and honor IPA’s dual hermeneutic. Participants were encouraged to provide specific real-life examples during the interviews to ground and enrich their accounts. No separate post-interview transcript-review or member-checking phase was conducted; the rationale for this is discussed below.

### Rigor and trustworthiness

Rather than being evaluated against criteria drawn from other qualitative traditions, this study’s methodological rigor was assessed using Yardley’s (2000; 2008) criteria for qualitative validity, the framework Smith et al. (2009) recommend for IPA research: sensitivity to context, commitment and rigor, transparency and coherence, and impact and importance. Sensitivity to context was addressed through purposive sampling of participants with direct, first-hand experience of the phenomenon under study and through prolonged engagement across 90–120 min interviews. Commitment and rigor were addressed through systematic, case-by-case immersion in each transcript prior to any cross-case comparison, consistent with IPA’s idiographic priorities. Transparency and coherence were addressed through the maintenance of reflexive memos documenting interpretive decisions and through a clear analytic pathway from raw transcript to emergent theme to superordinate domain. Consistent with IPA’s idiographic, single-analyst hermeneutic stance, this study did not employ triangulation across data sources, member checking, peer debriefing, independent or intercoder coding, or thematic saturation as validity checks; none of these are criteria specified by Smith et al. (2009) for evaluating IPA research, and each instead reflects assumptions about consensus, generalizability, or breadth-based confirmation drawn from other qualitative or quantitative traditions. Member checking in particular was not pursued because, under IPA’s double hermeneutic, a participant’s account may itself shift upon re-presentation – introducing an additional hermeneutic layer rather than confirming the original one – a rationale consistent with prior IPA research on posttraumatic growth (Smith et al., 2011). Saturation was likewise not pursued as a goal, since IPA’s aim is exhaustive, idiographic analysis of a small, purposively selected sample rather than breadth-based confirmation across cases.

### Reflexivity and researcher positionality

The author of this study also developed and previously reported on the culturally grounded intervention examined here (Varshney et al., 2022, 2024, 2025) and served as the sole analyst for the present IPA. This dual role of intervention developer and analyst is acknowledged as a meaningful source of potential confirmation bias: prior investment in the intervention’s value could plausibly have shaped how participant accounts were read and interpreted. Reflexive memos were kept throughout analysis in an effort to document interpretive decisions and to separate participants’ own language from the analyst’s interpretive gloss wherever possible. No independent or second-coder check was available to formally test this risk, however, and this is acknowledged as an unresolved limitation rather than a fully mitigated one (see “Limitations”).

## Results

IPA analysis yielded four superordinate experiential domains and nine subordinate themes, each supported by participant verbatims. While domains are conceptually distinct, participants’ experiences often overlapped; these were analytically separated for clarity (Table 3).

**TABLE 3:** The Superordinate and subordinate themes identified through IPA.

S. No	Super-ordinate themes	Sub-ordinate themes	Domain (1–4)
1	Psychological and Emotional Transformation	1.1 Emotional Liberation and Expression	Domain 1
		1.2: Rebuilding Identity, Confidence, and Future Orientation	Domain 1
		1.3: Meaning-Making Through Helping Others	Domain 1
2	Sociocultural and Systemic Trauma Context	2.1: Cultural Stigma, Silence, and Fear of Blame	Domain 2
		2.2 Unsafe Homes, Institutions, and Re-traumatizing Environments	Domain 2
3	Embodied and Relational Pathways to Healing	3.1: Embodied Recovery Through Breath-work, Grounding, and Spirituality	Domain 3 – Embodied & Relational
		3.2: Therapeutic Alliance and Safe Relational Holding	Domain 3
4	Non-linear Recovery and Ongoing Vulnerability	4.1: Episodic Relapse and Persistent Vulnerability	Domain 4
		4.2: Using New Coping Skills to Manage Triggers	Domain 4
5	Psychological and Emotional Transformation	5.1: Shifts in Perception of Self and Others	Domain 1
6	Sociocultural and Systemic Trauma Context	6.1: Stigma, Discrimination, and Community Neglect	Domain 2
		6.2: Family and Community Reactions as Barriers to Healing	Domain 2
7	Embodied and Relational Pathways to Healing	7.1: Relational Support as a Healing Anchor	Domain 3
8	Non-linear Recovery and Ongoing Vulnerability	8.1: Re-traumatisation Through Social Encounters	Domain 2
		8.2: Building Strength Through Avoidance of Unsafe Spaces	Domain 4
9	Psychological and Emotional Transformation	9.1: Growth in Inner Strength, Confidence, and Self-Belief	Domain 1
		9.2: Positive Reappraisal and Changing Self-Perception	Domain 2
10	Sociocultural and Systemic Trauma Context	10.1: New Environments as Protective Factors	Domain 2
11	Embodied and Relational Pathways to Healing	11.1: Embodied Coping: Breathwork, Meditation, Chanting, and Music	Domain 3
		11.2: Relational Sources of Strength	Domain 3
12	Non-linear Recovery and Ongoing Vulnerability	12.1: Inner Strength Developed Through Repeated Relapse Cycles	Domain 4
		12.2: Adaptive Solitude and Emotional Regulation	Domain 4
13	Psychological and Emotional Transformation	13.1: Emotional Relief and Renewed Hope Through Relational Support	Domain 1
		13.2: Strengthening of Self-Efficacy Through Guided Techniques	Domain 1
14	Sociocultural and Systemic Trauma Context	14.1: Contrast Between Healing Relationships and Past Harmful Authority Figures	Domain 2
		14.2: Community-Based and Spiritual Contexts Supporting Relational Healing	Domain 2
15	Embodied and Relational Pathways to Healing	15.1: Therapy as a Space for Embodied Co-Regulation	Domain 3
		15.2: Trust, Safety, and Validation Within the Therapeutic Alliance	Domain 3
16	Non-linear Recovery and Ongoing Vulnerability	16.1: Therapeutic Support During Relapse and Emotional Setbacks	Domain 4
		16.2 Integration of Therapeutic Skills Into Daily Life	Domain 4
17	Embodied Regulation and Physiological Stabilisation	17.1: Improved Breath Control and Reduction in Physiological Arousal	Domain 3
		17.2: Sensory Awareness and Grounding of the Body	Domain 3
18	Emotional Reorganization and Cognitive Shifts	18.1: Reduction in Rumination and Emotional Turbulence	Domain 1
		18.2: Strengthened Intuition and Emotional Discernment	Domain 1
19	Relational and Environmental Reconfiguration	19.1: Influence of Healing Environment on Emotional Regulation	Domain 2
		19.2: Dependence on Therapeutic Support During Setbacks	Domain 4
20	Emergence of Self-Empowerment and Identity Reconstruction	20.1: Growing Sense of Personal Agency and Self-Belief	Domain 1
		20.2: Integration of Healing Practices Into Daily Routines	Domain 1
21	Reframing Trauma Through Cognitive Insight and Mind Control	21.1: Trauma Reinterpretation Through Mental Discipline	Domain 1
22	Emotional Integration and Acceptance of the Past	22.1: Acknowledging the Permanence of Pain While Moving Forward	Domain 1
23	The Healing Role of Practice, Routine, and Consistency	23.1: Discipline, Routine, and Sustained Healing	Domain 1
24	Identity Reconstruction and Emergent Helper Identity	24.1: From Survivor to Helper – A Strengthened Identity	Domain 1
25	Integrating Knowledge and Experience (Cognitive + Embodied Understanding)	25.1: Bridging Scientific Understanding and Personal Experience	Domain 3
26	Emergence of Generativity and the Desire to Help Others	26.1: Providing Empathic Support and Normalizing Trauma	Domain 1
27	Teaching Skills and Strategies for Self-Healing	27.1: Promoting Meditation, Breathwork, and Focused Practices	Domain 3
28	Creating Safe Environments and Social Conditions for Recovery	28.1: Changing the Environment and Seeking Trusted Support	Domain 2
29	Empowerment and Encouraging Self-Reliance	29.1: Encouraging Inner Strength and the Will to Heal	Domain 1
30	Spiritual and Moral Guidance as a Pathway for Others	30.1: Faith, Prayer, and Moral Anchoring	Domain 3
31	Regaining Cognitive Clarity and Control	31.1: Strength Emerging Through Improved Focus and Mental Organization	Domain 1
32	Taking Decisive Action in Overwhelming Circumstances	32.1: Strength as Making a Life-Changing Choice	Domain 4
33	Embodied Realisation of Healing Through Practices	33.1: Physical Sensations of Relief as Signals of Strength	Domain 3
34	Growing Confidence and Self-Efficacy	34.1: Strength as Confidence in One’s Ability to Handle Life Independently	Domain 1
35	Spiritual or Existential Turning Points	35.1: Strength Emerging From Spiritual Pathways	Domain 2
36	Cognitive Reframing and Change in Outlook	36.1: Healing as a Shift in Perception Toward People and Life	Domain 1
37	Emotional Letting Go and Moving Beyond the Past	37.1: Healing as the Ability to Move On From Traumatic Memories	Domain 1
38	Somatic and Cognitive Sensitivity to Social Reactions	38.1: Healing as Stability That Can Be Disrupted by Stigma or Labeling	Domain 2
39	Emergence of Assertiveness and Agency	39.1: Healing as the Ability to Speak Up, Take a Stand, and Protect Others	Domain 1
40	Healing as Autonomy From Medication and Restoration of Cognitive Function	40.1: Recognizing Healing Through Improved Memory, Focus, and Reduced Dependency	Domain 1
41	Healing as Growth, Maturity, and Life Wisdom	41.1: Learning From Negative Experiences	Domain 1

### Domain 1: psychological and emotional transformation

This domain captures the intrapsychic shifts that enabled participants to move from emotional blockage, fractured identity, and overwhelming symptoms toward emotional openness, confidence, and future-oriented agency.

*1.1 Emotional liberation and expression*

**P1:** *“Earlier I was unable to share and express my trauma and emotions inside me… grounding, centering and meditation provided so much relief… After following meditation all these years, I’m able to share my emotions with everyone.”*

**P2:** *“Earlier I was seriously depressed and traumatized… now I am more at peace… I have learnt to cope up with life challenges on my own.”*

**P3:** *“I learnt Pranayama and meditation and it greatly benefited me… I feel I am more confident now.”*

Before intervention, all three participants struggled with suppressed emotions and internalized distress. After therapy, they reported a newfound ability to articulate emotions and regulate distress. This emotional liberation was centrally tied to breathwork and grounding practices. IPA interpretation identifies this as the reclaiming of emotional agency in a cultural context where trauma expression is discouraged and silence is normative.

*1.2 Identity reconstruction and future orientation*

**P1:** *“I see myself as someone happy and enjoying life… I plan to do MBA in health management… I also started an online business.”*

**P2:** *“I fulfill my childhood aspiration through the spiritual discourse training… I know I cannot go back to my previous toxic environment.”*

**P3:** *“I feel I am progressing well in life and career… I am more confident.”*

Participants described significant shifts in self-concept and future orientation. P1’s educational and entrepreneurial aspirations, P2’s spiritual fulfillment, and P3’s integration of motherhood and career reflect a reconstructed sense of purpose and agency. IPA interpretation suggests a shift from trauma identity to empowerment identity, a core component of posttraumatic growth (PTG).

*1.3 Altruistic growth and meaning-making*

**P1:** *“I’m also sharing my practices with other patients… I try to help whenever I can.”*

**P2:** *“I cope by learning and helping others in the spiritual place.”*

**P3:** *“I support my husband and child… and others who are struggling.”*

A consistent pattern was the emergence of altruistic action, wherein participants used their personal suffering as motivation to help others. This reflects the PTG dimension of relational and prosocial growth. Researchers interpret this as survivors translating suffering into service, a meaningful outcome in collectivist tribal communities.

### Domain 2: Sociocultural and systemic trauma context

This domain captures how trauma in tribal Sikkim is embedded in structural inequalities, patriarchal norms, caste-based discrimination, and institutional neglect rather than isolated adverse events.

*2.1 Cultural stigma, silence, and fear of blame*

**P1:** *“I was too scared and thought I would be blamed… I could not share with even close family members.”*

**P2:** *“My previous home environment was very toxic… I keep reliving the same moments at home which traumatizes me.”*

**P3:** *“I could not adjust in my in-laws’ home… fear and anxiety increased again.”*

Participants’ narratives reveal deep entanglement of trauma with cultural stigma and fear of blame. Sociocultural forces created an atmosphere of emotional suppression and discouraged disclosure, intensifying distress. Researchers interpret this silence not as passivity but as self-protection within patriarchal, stigma-laden environments where emotional expression by women carries significant social cost.

*2.2 Re-traumatizing environments and structural barriers*

**P1:** *“The private university had a toxic environment… I was admitted to hospital after ragging-this was a relapse.”*

**P2:** *“I don’t stay at home… it keeps me traumatized… I avoid going back.”*

**P3:** *“My mother-in-law’s strict nature triggered my anxiety… I relapsed.”*

Trauma was perpetuated not only by past events but by ongoing exposure to unsafe educational spaces, dysfunctional families, and oppressive marital homes. Caste-based discrimination from institutional authority figures added unique identity-based layers of trauma rarely described in dissociative disorder literature. IPA interpretation frames these as structural trauma, where caste, hierarchy, and patriarchal systems intensify and sustain psychological suffering.

### Domain 3: embodied and relational pathways to healing

This domain explains how healing occurred through somatic regulation, therapeutic alliance, relational safety, and spiritual community-the primary bio-behavioral mechanisms of symptom reduction.

*3.1 Embodied recovery through breathwork, meditation, and music*

**P1:** *“When I get triggers, I follow breathwork and get fast relief.”*

**P2:** *“The spiritual routine gives me peace… fulfills my childhood aspiration.”*

**P3:** *“Pranayama and meditation helped my panic attacks and memory lapses… the same therapy helped again during relapse.”*

Breathwork, grounding, and meditation provided moment-to-moment physiological regulation. Participants described “fast relief,” “slowing of thoughts,” and “feeling refreshed,” suggesting parasympathetic activation and vagal engagement. The efficacy of breathwork in trauma management is well established (Fonkoue et al., 2020; Jerath et al., 2006; Seppala et al., 2014). Raga-based music therapy engaged rhythmic regulation mechanisms, consistent with psychophysiological models of somatic healing. Unlike Western somatic models such as Trauma-Sensitive Yoga, which depend on structured groups and cultural familiarity with yoga, these practices were indigenous and deeply embedded in participants’ sociocultural worlds.

*3.2 Therapeutic alliance and relational safety*

**P1:** *“I could fully disclose to someone I could connect with… this gave immense relief.”*

**P2:** *“I feel supported in the spiritual space… I feel understood.”*

**P3:** *“My fiancé accompanied me… I benefitted more.”*

Healing was centrally facilitated by relational safety-trust, connection, and acceptance. Therapists, trusted partners, and spiritual teachers served as anchoring figures enabling open trauma processing. Participants’ descriptions of “full disclosure,” “felt understood,” and “helped me understand my symptoms” mirror key components of trauma-informed care: attunement, containment, and empowerment. IPA interpretation frames this as the relational co-regulation essential in collectivist cultures where healing is socially distributed rather than individually generated (Norcross and Lambert, 2018).

### Domain 4: non-linear recovery and ongoing vulnerability

Recovery was cyclical, not linear. Stressors triggered relapses, but internalized coping skills prevented regression to prior severity.

*4.1 Episodic relapse and persistent vulnerability*

**P1:** “*This was a relapse after 3–4 years… but I continued my practices.”*

**P2:** *“Sometimes I have negative thoughts but I cope before it gets worse.”*

**P3:** *“I relapsed after marriage… anxiety, panic attacks returned.”*

All participants experienced symptom recurrence during stressful life events. However, relapse episodes did not escalate to previous severity, demonstrating increased internal resilience. IPA interpretation frames relapse not as failure but as a developmental site of skill consolidation, consistent with dynamic models of trauma recovery (Hayes et al., 2007).

*4.2 Adaptive coping and environmental restructuring*

**P1:** *“When I get the triggers, I follow breathwork and get fast relief… My new environment is much better.”*

**P2:** *“I moved out of my home to escape… I cope with my negative thoughts before they worsen.”*

**P3:** *“I relocated and live closer to my workplace… the same therapy helped me again.”*

Participants actively used learned coping strategies-breathwork, meditation, adaptive solitude, and strategic relocation from harmful environments. Physical distancing from toxic spaces acted as a protective behavioral restructuring strategy rather than mere avoidance. This finding aligns with trauma-informed care literature identifying safe environments as crucial mechanisms for preventing retraumatization (Goldstein et al., 2024; Owen and Crane, 2022).

### The TRIBE-HEAL model

**FIGURE 1:**
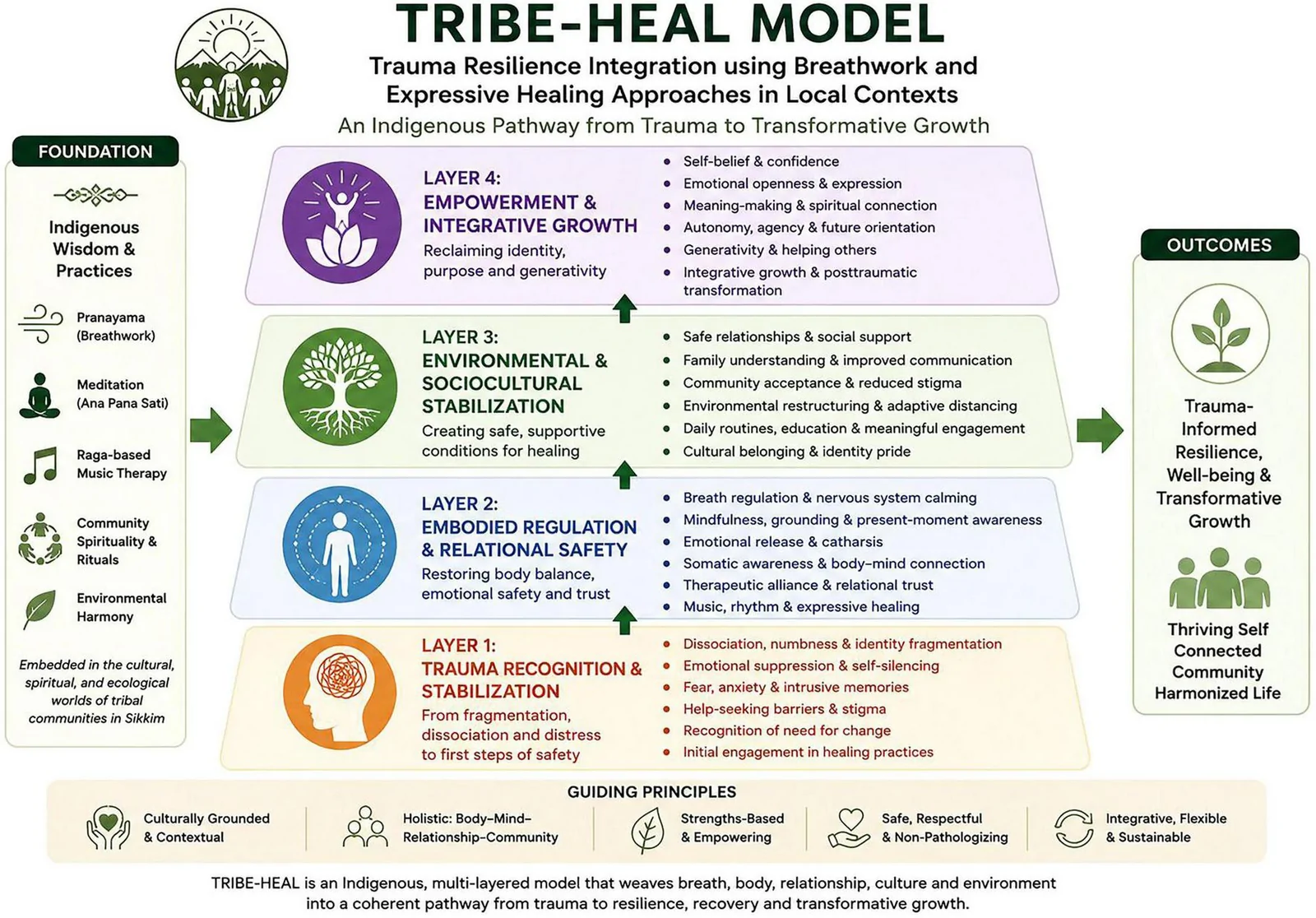
The TRIBE-HEAL model.

As shown in Figure 1, the four experiential domains and their subordinate themes collectively informed TRIBE-HEAL (Trauma Resilience Integration using Breathwork and Expressive Healing Approaches in Local contexts), a culturally grounded behavioral medicine framework for trauma recovery in marginalized tribal settings. Each model layer corresponds directly to healing mechanisms identified in participant narratives.

*T – Trauma Recognition and Reframing* (Cognitive, emotional, and identity-based reorganization, corresponding to Domain 1) captures the intrapsychic reconstruction from fragmented self-states to coherent self-awareness. Through psychoeducation, therapy, and mindfulness, participants named, located, and reinterpreted their trauma. Cognitive reframing reduced catastrophic thinking; identity reconstruction enabled the transition from victimhood to helper identity, an advanced form of PTG.

*R – Relational Safety and Regulation* (Therapeutic alliance and community healing structures, corresponding to Domain 3.2) highlights that for these women, safety was cultivated in the presence of safe others. Therapeutic co-regulation, consistent with polyvagal theory, downregulated defensive physiology. Spiritual and community spaces extended this safety, while unsafe authority figures and critical family responses underscore why relational safety must be consciously built.

*I – Interoceptive and Embodied Regulation* (Breathwork, grounding, sensory awareness, corresponding to Domain 3.1) represents the experiential core of the model. Participants described breathwork as producing what they experienced as immediate physiological downregulation, grounding techniques as increasing body awareness, and chanting and music as engaging a sense of rhythmic regulation. Participants described somatic markers of healing (“my body became light,” “heat left the body”), which is interpretively consistent with, though not directly evidenced by, models of vagally mediated stabilization, as no physiological measures were collected in this study.

*B – Belief Systems, Spiritual Anchors, and Meaning-Making* (Corresponding to Domain 3.1 and Domain 1) highlights the role of indigenous spiritual practices, breathwork-based rituals, and meaning-making in sustaining recovery. For these participants, healing was inseparable from spiritual belief: Pranayama, mantra chanting, raga music, and prayer were not merely techniques but acts of faith embedded in cultural identity. Spiritual routines offered predictability, moral anchoring, and existential meaning-vital healing mechanisms in rural tribal health contexts. Participants described breathwork and spiritual practice as their first-line response to triggers, reflecting deep internalization of culturally congruent regulatory beliefs.

*E – Environmental Restructuring for Safety* (Derived from Domains 2 and 4) captures the role of physical and relational environment in sustaining recovery. Participants who relocated from toxic households, changed institutions, or entered spiritually supportive settings reported significant emotional stabilization. This layer recognizes that internal regulation alone is insufficient when structural environments continuously reactivate trauma-healing therefore requires deliberate environmental change.

*H – Healing as Non-Linear and Skill-Reinforcing* (Corresponding to Domain 4) reflects the cyclical nature of recovery. Relapse was not failure but a site of skill consolidation. Each recurrence of symptoms provided an opportunity for participants to apply and deepen their regulatory practices, gradually building resilience that reduced the intensity and duration of subsequent episodes.

*E – Empowerment and Identity Rebuilding* (Extending Domain 1) is the growth layer where resilience becomes enacted in daily life. Participants showed increasing agency, autonomy from external control, new career and educational goals, and a culturally meaningful empowerment through helping others. The shift from trauma identity to helper identity-a form of generative posttraumatic growth-was a defining outcome across all three narratives.

*A – Altruistic Growth and Generativity* (Emerging from Domain 1) captures the prosocial dimension of recovery. All three participants described using their own healing experience to support peers, community members, or family. This altruistic orientation reflects a culturally congruent form of PTG wherein meaning is made not through private reflection but through relational service-a pathway particularly significant in collectivist tribal communities.

*L – Long-Term Cognitive and Emotional Integration* (Meta-level synthesis layer integrating all four domains) represents the sustained consolidation of all prior healing mechanisms into a coherent, lived practice. Participants described using breathwork automatically upon triggering, maintaining spiritual and creative routines, viewing themselves as capable and deserving, and reinterpreting setbacks through a growth lens. Rather than a fixed endpoint, integration is an ongoing biopsychosocial-spiritual process-consistent with behavioral medicine models of chronic condition self-management.

Unlike Western somatic models such as Trauma-Sensitive Yoga (TSY/TCTSY), which have demonstrated benefits for PTSD but depend on structured groups, certified facilitators, and cultural familiarity with Western mindfulness (Cramer et al., 2018; West et al., 2017), TRIBE-HEAL arises directly from the lived experiences of tribal adolescent girls and young women with dissociative disorders. It integrates indigenous practices-pranayama, raga-based music, community spirituality, and environmental restructuring-embedded in their sociocultural world and is therefore contextually emergent rather than an adaptation of imported frameworks.

This model extends the scientific arc established across three previous studies from Sikkim: the first documenting how misdiagnosis and cultural stigma obstruct care pathways for dissociative girls (Varshney et al., 2022); the second uncovering cognitive and emotional mechanisms-self-silencing, rumination, and identity fragmentation-in traumatized girls (Varshney et al., 2024); and the third providing early clinical evidence that breathwork stabilizes physiological arousal and reduces dissociative episodes (Varshney et al., 2025). TRIBE-HEAL synthesizes these prior insights with new IPA-derived data to propose a comprehensive, culturally responsive trauma-recovery framework that is biologically plausible, culturally resonant, and behaviorally actionable.

## Discussion

This study contributes several important insights to the literature on trauma recovery, dissociative disorders, and culturally grounded behavioral health. The findings are discussed in relation to existing theory and research across the four experiential domains.

### Posttraumatic growth in marginalized tribal contexts

Participants described experiences reflecting all five core dimensions of posttraumatic growth (PTG) identified by Tedeschi et al. (2018): new possibilities, relating to others, personal strength, spiritual change, and appreciation of life. Consistent with a body of IPA research examining PTG entirely through interview-based, idiographic accounts rather than a validated psychometric scale such as the Posttraumatic Growth Inventory (Smith et al., 2011; Sheridan and Carr, 2020; Kaye-Tzadok and Icekson, 2022), PTG in this study is presented as a qualitatively described experience rather than a quantitatively measured outcome, and no claim of a validated or standardized PTG assessment is made. Notably, their PTG unfolded through indigenous body-mind practices rather than Western psychotherapeutic exposure. This is consistent with emerging scholarship showing that PTG in non-Western, collectivist communities is frequently mediated through spiritual meaning-making, relational belonging, and embodied cultural rituals rather than cognitive restructuring alone (Gee et al., 2023). The altruistic orientation observed across all three participants – sharing practices with peers, supporting community members, assuming helper roles – reflects a culturally congruent form of prosocial PTG rarely foregrounded in Western trauma models (Canevello et al., 2022).

Importantly, PTG in this sample coexisted with ongoing vulnerability and relapse. Rather than representing a linear endpoint, growth emerged iteratively through cycles of challenge, coping, and re-stabilization. This reinforces critiques of stage-based recovery models and supports dynamic, non-linear frameworks for understanding trauma outcomes in structurally marginalized populations (Hayes et al., 2007).

### Sociocultural and structural dimensions of trauma

A key finding of this study is that trauma in tribal Sikkim cannot be understood as a purely psychological phenomenon. Participants’ dissociative symptoms were embedded in, and perpetuated by, structural inequalities: caste-based discrimination, patriarchal family systems, gendered silencing, unsafe educational institutions, and weak community mental health infrastructure. This aligns with Subica and Link’s (2022) formulation of cultural trauma as a fundamental cause of health disparities and with scholarship on caste-based trauma among Dalit and Adivasi communities in India (Mehta, 2025; Gupta and Coffey, 2020).

Particularly noteworthy is the role of unsafe environments not only as antecedents to disorder onset but as active agents of relapse. All three participants experienced re-traumatization through ongoing contact with toxic households, institutional spaces, or community actors. This finding supports calls for trauma-informed care frameworks to extend beyond the therapeutic encounter to include environmental safety planning, safe housing pathways, and systemic advocacy (Goldstein et al., 2024; Decker et al., 2022). Healers and clinicians working in these communities must address structural determinants alongside psychological symptoms if interventions are to produce durable change.

### Embodied and relational healing mechanisms

The centrality of breathwork, meditation, and raga-based music therapy in participants’ recovery is consistent with a substantial body of evidence on somatic and breath-based trauma interventions. Slow diaphragmatic breathing modulates vagal tone and sympathetic arousal (Jerath et al., 2006; Fonkoue et al., 2020), while rhythmic auditory stimulation through music engages predictable neurological regulation pathways (Kim et al., 2013). Participants described these effects somatically – “fast relief,” “heat leaving the body,” “head clearing” – suggesting genuine neurophysiological downregulation rather than placebo or expectation effects. A recent meta-analysis confirmed the effectiveness of pranayama for mental health across randomized controlled trials (Mütze et al., 2025), and prior research from Sikkim has established its clinical utility in dissociative disorder management (Varshney et al., 2025).

Relational safety was an equally central mechanism. Consistent with polyvagal theory (Porges, 2011) and trauma-informed relational models (Stanley, 2016), participants described healing as occurring in co-regulated dyads and community contexts – with therapists, spiritual teachers, trusted relatives, and fiancés serving as anchoring figures. The therapeutic alliance has been identified as among the most robust predictors of trauma therapy outcomes across modalities (Norcross and Lambert, 2018; Burback et al., 2025), and these findings reinforce its particular salience in collectivist tribal settings where healing is understood as relational and communal rather than individual.

What distinguishes this study’s findings from Western somatic models is the seamless integration of breathwork with spiritual practice, community ritual, and musical tradition. Participants did not experience Pranayama and raga music as clinical techniques applied by an expert but as extensions of their existing cultural repertoire-practices that already carried meaning, social recognition, and existential significance. This cultural embeddedness likely enhanced both the acceptability and the sustained practice of these modalities long after formal intervention ended.

### Non-linear recovery and the role of skill integration

The non-linear recovery trajectories observed in this study align with dynamic systems perspectives on trauma healing, which emphasize that recovery involves iterative cycles of destabilization and reorganization rather than unidirectional symptom reduction (Hayes et al., 2007). All three participants experienced meaningful relapses triggered by environmental stressors, yet none regressed to pre-intervention severity. This pattern is consistent with participants describing themselves as having developed durable internal resources-interoceptive awareness, emotional regulation capacity, and behavioral coping strategies-that they experienced as moderating the impact of ongoing adversity; as a qualitative, idiographic study, this cannot establish that the intervention caused these resources to develop.

Critically, participants integrated therapeutic skills into daily routines without ongoing clinical support. Breathwork upon triggering, raga music for emotional regulation, meditation for rumination management, and strategic withdrawal from unsafe spaces became autonomous self-care behaviors. This naturalistic generalization of skills is a particularly important outcome in low-resource settings where continuous access to trained mental health professionals is unavailable. It points to the scalability of interventions that equip individuals with portable, culturally congruent regulatory tools rather than depending on sustained provider contact.

### Limitations

Several limitations must be acknowledged. The sample comprises three participants, which, while appropriate for IPA and consistent with its epistemological commitments (Smith et al., 2009), restricts the transferability of findings. These are not claims to statistical generalization but to theoretical and conceptual illumination-insights about the structure and texture of healing in this specific context that may resonate in comparable settings. The single-researcher design, while mitigated by reflexive memos and participant examples, introduces the risk of interpretive bias. This risk is compounded by the fact that the author also developed the intervention under study, creating a dual role (developer and analyst) that may have predisposed the analysis toward confirmatory interpretations; this is discussed further under Reflexivity and Researcher Positionality above. Relatedly, and consistent with IPA’s own validity criteria rather than those of other qualitative traditions, this study did not employ triangulation, member checking, peer debriefing, independent coding, or saturation as markers of rigor (see Rigor and Trustworthiness above); readers should weigh the findings with this methodological framing in mind. The use of follow-up interviews at a single time point also limits understanding of how healing continues to evolve. Interviews were conducted approximately 3 years after the intervention concluded, and retrospective accounts of this kind are subject to memory reconstruction over time; while IPA is concerned with how participants currently make sense of past experience rather than with securing an objectively accurate historical record, the extended recall interval should be considered when weighing the specificity of participants’ accounts. In addition, somatic and embodied descriptions in this study are interpreted in relation to neurophysiological concepts such as vagal regulation for conceptual context only; no physiological measures (e.g., heart-rate variability, cortisol) were collected, and such interpretations should be read as illustrative rather than as physiological evidence. Future research employing larger samples, multiple coders, and longitudinal designs is necessary to examine the stability and broader applicability of these findings and the TRIBE-HEAL model.

## Conclusion

This study examined how tribal adolescent girls and young women with dissociative disorders understand their healing and posttraumatic growth following a culturally grounded breathwork and raga-based intervention. IPA illuminated four interrelated experiential domains-psychological-emotional transformation, sociocultural and systemic trauma context, embodied and relational healing mechanisms, and non-linear resilience-revealing a multidimensional, culturally embedded pathway through which trauma recovery unfolds in marginalized tribal settings.

Participants described marked psychological shifts: reduced fear, improved emotional regulation, restored confidence, and identity reconstruction. These changes were rooted in breathwork, music, and spiritual anchoring-modalities deeply embedded in their sociocultural milieu rather than imposed from Western frameworks. Persistent stigma, unsafe households, rigid gender norms, and institutional neglect emerged as structural contributors to both the onset and maintenance of dissociation, underscoring that healing in these communities cannot be understood without acknowledging the cultural and environmental constraints shaping vulnerability.

Embodied and relational regulation were central mechanisms. Pranayama, raga-based music, and meditation directly calmed the body, reduced physiological arousal, and interrupted dissociative spirals. Relational safety-through therapists, trusted relatives, peers, or spiritual communities-functioned as a parallel regulation system. Recovery trajectories were non-linear, with relapse serving as a site of skill consolidation rather than failure.

The TRIBE-HEAL model, the first trauma-informed framework grounded in the lived experience of tribal young women with dissociative disorders in India, synthesizes these findings across cognitive reframing, relational co-regulation, interoceptive stabilization, behavioral adaptation, environmental restructuring, and long-term integration. It offers clinicians, researchers, and policymakers a multidimensional, low-cost, scalable roadmap for community-based behavioral health in underserved populations.

Future research should examine TRIBE-HEAL across diverse minoritized groups, employ physiological markers (HRV, cortisol) and neuroimaging to validate mechanistic pathways, and test structured intervention modules derived from the model. Longitudinal and mixed-method designs are critical for assessing stability, generalizability, and health-system integration.

## Data Availability

The original contributions presented in the study are included in the article, further inquiries can be directed to the corresponding author.
